# Oestrogenic pollutants promote the growth of a parasite in male sticklebacks

**DOI:** 10.1016/j.aquatox.2016.02.010

**Published:** 2016-05

**Authors:** Vicki Macnab, Ioanna Katsiadaki, Ceinwen A. Tilley, Iain Barber

**Affiliations:** a Department of Neuroscience, Psychology and Behaviour, College of Medicine, Biological Sciences and Psychology, University of Leicester, Leicester LE1 7RH, UK; b Cefas Weymouth Laboratory, The Nothe, Weymouth DT4 8UB, UK

**Keywords:** Endocrine disruption, Parasitism, Pollution, Disease, Infection phenotype, Oestrogens

## Abstract

Aquatic environments are especially susceptible to anthropogenic chemical pollution. Yet although knowledge on the biological effects of pollutants on aquatic organisms is increasing, far less is known about how ecologically-important interspecific interactions are affected by chemicals. In particular, the consequences of anthropogenic pollution for the interaction of hosts and parasites are poorly understood. Here, we examine how exposure to 17β-oestradiol (E2)—a natural oestrogen and a model endocrine disrupting chemical (EDC) —affects infection susceptibility and emergent infection phenotypes in an experimental host–parasite system; three spined sticklebacks (*Gasterosteus aculeatus*) infected with the common, debilitating cestode *Schistocephalus solidus*. We exposed individual sticklebacks to a 0 ng l^−1^ (control), 10 ng l^−1^ or 100 ng l^−1^ E2 treatment before feeding them infective stages of *S. solidus*. E2 exposure significantly elevated vitellogenin (VTG) levels—a biomarker of exposure to xenoestrogens—in both female and male fish, and reduced their body condition. Susceptibility to parasite infection was unaffected by EDC exposure; however, E2 treatment and fish sex interacted significantly to determine the growth rate of parasites, which grew quickest in male hosts held under the higher (100 ng l^−1^) E2 treatment. Tissue VTG levels and parasite mass correlated positively across the whole sample of experimentally infected fish, but separate regressions run on the male and female datasets demonstrated a significant relationship only among male fish. Hence, among males—but not females—elevated VTG levels elicited by E2 exposure led to more rapid parasite growth. We outline plausible physiological mechanisms that could explain these results. Our results demonstrate that oestrogenic pollutants can alter host–parasite interactions by promoting parasite growth, and that male hosts may be disproportionately affected. Because ecologically-relevant effects of infection on host antipredator responses, growth, energetics and reproductive development all depend on parasite mass in this host–parasite system, our results indicate that EDCs can mediate the ecological consequences of infections. We therefore consider the implications of our results for the ecology of hosts and parasites in polluted environments.

## Highlights

• E2 exposure had no effect on the susceptibility of sticklebacks to parasite infection.
• E2 elevated VTG levels in males and females.
• E2 increased the growth of parasites in male, not female, fish.
• Parasite mass correlated with VTG levels among males, but not females.

## 1 Introduction

Human activities are rapidly changing the conditions that wildlife experience in nature (Candolin and Wong, 2012, Dubinsky and Stambler, 1996, Harvell et al., 2002). As a consequence, organisms already encountering a range of natural environmental stressors, including predators, parasites and competitors, are additionally faced with a broad array of anthropogenic stressors. In the laboratory, the effects of individual natural and anthropogenic stressors are typically studied in isolation, with test organisms otherwise experiencing ideal conditions (Holmstrup et al., 2010, Sures, 2008). However, environmental stressors have considerable capacity to interact and produce additive, synergistic or antagonistic effects (Brian et al., 2005, Kiesecker, 2002, Marcogliese et al., 2010), and understanding how multiple stressors interact to influence animal health in anthropogenically disturbed ecosystems represents a key challenge (Marcogliese and Pietrock, 2011, Sih et al., 2004). In particular, there is a need for a far greater understanding of how exposure to anthropogenic stressors affects the capacity of individual organisms to cope with the perennial latent threat imposed by parasitic infections (Lafferty and Kuris, 1999, Lafferty and Kuris, 2005).

Chemical pollution represents one of the biggest threats to animal health in natural ecosystems (Martin et al., 2010), and chemical input via domestic and industrial effluents, and through agricultural runoff, means that aquatic habitats are particularly threatened (Sumpter, 2009). Chemical pollutants can be a contributing factor in disease outbreaks and can influence host–parasite interactions directly, e.g. through toxic effects on intermediate hosts or parasite themselves, or indirectly, e.g. through suppressive effects on host immune systems (Dobson and Foufopoulos, 2001, King et al., 2010, Martin et al., 2010, Poulin, 1992). Parasite infections can have unpredictable effects on host responses to chemical pollution; for example, some acanthocephalan worms sequester heavy metal pollutants from the tissues of host fish such that infected fish accumulate lower concentrations than non-infected conspecifics (Sures, 2008). Since fish are likely to encounter both parasites and chemical pollutants in their natural environments, they represent ideal vertebrate models for studying interactions between anthropogenic and natural stressors.

Endocrine disrupting chemicals (EDCs) represent a particularly important group of pollutants affecting the reproductive potential of fish and other aquatic organisms (Jobling et al., 1998). EDCs can act both agonistically, by mimicking natural hormones, or antagonistically, by binding to hormone receptors and blocking responses, and thus have considerable potential to interfere with normal reproductive development (Colborn et al., 1993, Jobling et al., 1998, Tyler et al., 1998). Known endocrine disruptors include natural steroids that can persist in sewage effluent after treatment, synthetic xenoestrogens (including pharmaceuticals), components used in pesticides, and plasticisers—such as phthalates—that have oestrogenic activity (Purdom et al., 1994, Thomas et al., 2002).

We tested the effect of the natural steroid 17β-oestradiol (E2) on disease susceptibility and progression in the stickleback-*Schistocephalus* experimental model system, which is ideally suited for studying host–parasite interactions under changing environments (Barber, 2013, Barber and Scharsack, 2010, Macnab and Barber, 2012). The three-spined stickleback *Gasterosteus aculeatus* is well-established as a model species in the field of endocrine disruption (Katsiadaki et al., 2007), and E2 was selected as representative of a large number of environmental EDC pollutants that have oestrogenic modes of action (Jobling et al., 1998, Purdom et al., 1994) and potential immunosuppressive effects (Milla et al., 2011). Sticklebacks naturally become infected with plerocercoid larvae of the diphyllobothriidean cestode *Schistocephalus solidus* after feeding on parasitized copepods, and the life cycle of the parasite is completed when infected sticklebacks are eaten by a susceptible definitive host, usually a piscivorous bird (Barber and Scharsack, 2010). Plerocercoids can grow to a large size, exerting significant energetic drain on host resources and negatively affecting stickleback growth and reproductive development (Heins et al., 2010, Rushbrook et al., 2007, Tierney et al., 1996). Larger plerocercoids impact more significantly on the reproductive potential (Heins et al., 2010, Macnab et al., 2009, Macnab et al., 2011) and the anti-predatory behaviour of host fish (Barber et al., 2004, Milinski, 1985). As such, *S. solidus* infections can have significant ecological implications, since they determine the reproductive potential of sticklebacks, and also direct predator-prey interactions of host fish.

We used the stickleback-*Schistocephalus* model to test the hypothesis that oestrogenic EDCs can affect the progression of parasitic disease in fish. Lab-bred fish were exposed continuously to either a solvent control or to a low (10 ng l^−1^) or a high (100 ng l^−1^) concentration of E2 for 18 days before being subject to experimentally parasite challenge. Fish were then placed back in their exposure groups for a further four week period, allowing us to address the following key questions: (1) does EDC exposure influence susceptibility to parasite infection, and (2) does EDC exposure affect parasite growth rate? By using molecular techniques to assign genetic sex to sexually immature host fish, we were additionally able to ask (3) does host sex modulate the effects of EDCs on these host–parasite interactions?

## 2 Methods

### 2.1 Experimental design

**Fig. 1:**
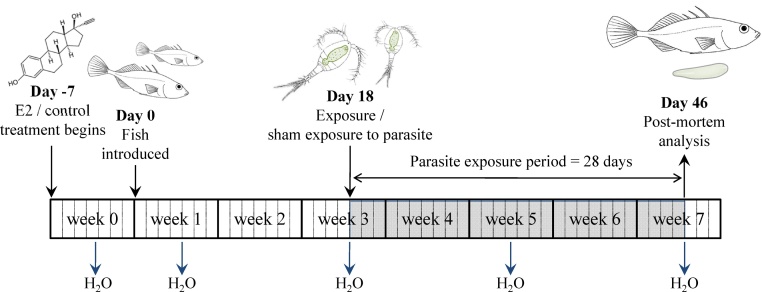
Schematic diagram showing the design of the experimental E2 treatment and parasite exposure study. Time points at which sticklebacks were exposed to E2 (or solvent control) and at which they were fed infective *Schistocephalus solidus* parasites are indicated. The shaded bar shows the period of parasite infection, whilst water sampling time points, for determination of E2 exposure levels, are also shown.

Juvenile three spined sticklebacks from fourteen families, generated by IVF (Barber and Arnott, 2000) using wild caught parents from Carsington Reservoir (UK: N53°03′21′′, W1°37′25′′), were reared in family groups in laboratory aquaria for 24 weeks. A total of 222 fish from these families were then distributed randomly between 27 flow-through test aquaria (each 10 l, 15 cm × 25 cm × 30 cm) such that each contained a group of 8–9 fish. Each test aquarium was then exposed to either a 10 ng l^−1^ (‘low’) or 100 ng l^−1^ (‘high’) E2 treatment, or to a solvent control treatment, with nine replicate aquaria per treatment. Target concentrations were achieved by mixing E2 stock solutions, prepared in EtOH and dissolved in dH_2_0, with a rate-regulated supply of copper-free, dechlorinated tap water that ensured three water changes per tank per day. The flow-through system was run for a minimum of 7 days prior to fish introduction, to allow target concentrations to stabilise (Sebire et al., 2008). Concentrations of E2 in the aquaria were determined at various time points during the exposure period (see below and Fig. 1).

### 2.2 Experimental infections of copepods and sticklebacks

After 18d of exposure to E2 or solvent control, all fish were subject to a controlled challenge with infective *S. solidus* parasites (Fig. 1). Plerocercoids dissected from naturally infected wild-caught Carsington Reservoir sticklebacks were cultured in vitro to stimulate egg production (Macnab and Barber, 2012). Eggs were incubated in the dark for 21 days before being exposed to natural light to stimulate hatching. Individual laboratory reared copepods (*Cyclops strenuus abyssorum*) were placed in a drop of water with 1–2 hatched coracidia in a Petri dish. Copepods were screened for infection after 10 days and those harbouring infective procercoids were fed to test sticklebacks (Macnab and Barber, 2012) before being placed back in their treatment tanks for a further 28 days. Fish were held at 18 ± 1.5 °C under a photoperiod of 16L:8D and fed daily, ad libitum, with frozen bloodworms (*Chironomus* sp. larvae) during the study.

### 2.3 Post mortem analyses

Mortalities (19 fish across the whole experiment, 8.6%) were evenly spread across treatment groups, generating sample sizes at the end of the study of *N* = 68 fish in the solvent control group, *N* = 66 in the low E2 group and *N* = 69 in the high E2 group. Surviving fish were killed using a lethal dose of Benzocaine anaesthetic, blotted to remove surface moisture, measured (standard length, *L*_s_, to 0.1 mm) and weighed (wet mass, *M*, to 0.001 g). A fin clip was placed in 1 ml EtOH for molecular determination of genetic sex. Each fish was then frozen at −20 °C for 30 days, after which the head and tail of each fish was removed, weighed and stored at −20 °C for analyses of tissue vitellogenin (see below). Phenotypic sex, infection status and the wet mass of any plerocercoids (*M*_p_, to the nearest μg) were also recorded. Body condition factor (*K* = 10^5^ [(*M* − *M*_p_) / *L*_s_^3^]) was also calculated as an index of energetic status (Pennycuick, 1971).

### 2.4 Vitellogenin (VTG) analyses

The egg yolk protein precursor, vitellogenin (VTG) is a widely-used biomarker of oestrogenic exposure in fish (Sumpter and Jobling, 1995). To quantify the effects of E2 exposure on tissue VTG levels we employed an in-house homologous ELISA, which has been operational for a decade (e.g. Hahlbeck et al., 2004, Katsiadaki et al., 2012, Katsiadaki et al., 2002) and recently validated as part of an international test guideline programme (OECD, 2011b). Briefly, head and tail samples were pooled for each fish and homogenisation buffer (50 mM Tris–HCl pH 7.4, 1% protease inhibitor cocktail [Sigma, UK]) was added. Tissue samples were homogenised with disposable pestles and immediately centrifuged at 13,500 rpm (1600 *g*) at 4 °C for 15 min. The supernatant was stored at −20 °C before determining the VTG content (OECD, 2011a).

### 2.5 Water sampling and radioimmunoassay (RIA)

Water samples (250 ml) were collected from the outflow of each tank during week 0, and on four further occasions over the course of the study to determine the actual chemical exposure concentration achieved (Fig. 1). E2 was extracted after pumping the water though Sep-pak Plus C18 solid phase extraction cartridges (Waters Ltd., UK). This method has been developed at the Cefas Weymouth Laboratory for a number of natural and synthetic steroids, including cortisol (Ellis et al., 2004), ethinyl-oestradiol (Katsiadaki et al., 2010), 11-ketotestosterone and E2 (Sebire et al., 2007). Briefly, each cartridge was primed with 5 ml of methanol followed by 5 ml of dH20 prior to pumping, and each cartridge was then washed with 5 ml of dH20 before being air-dried, wrapped in Parafilm^®^ and stored at −20 °C until elution of E2. After eluting E2 from the cartridges, its concentration was quantified using a radioimmunoassay (RIA) with a detection limit of 0.5 ng ml^−1^ (Scott et al., 1984, Sebire et al., 2007).

E2 concentrations in test aquaria were close to nominal levels; over the exposure period the mean (±SE) concentration achieved was 7.8 ± 0.49 ng l^−1^ in the 10 ng l^−1^ treatment and 65.5 ± 4.82 ng l^−1^in the 100 ng l^−1^ treatment. Levels of E2 in the solvent control were negligible (0.8 ± 0.05 ng l^−1^). Data were not corrected for recovery rates, so values include any losses during the extraction procedure. Based on our laboratory’s extensive experience, typical recovery rates for steroidal oestrogens during this extraction procedure are between 10–30%.

### 2.6 Molecular sex determination

DNA was extracted from pectoral fin clips using isopropanol precipitation, based on published protocols (Sambrook and Russell, 2001). The samples were then genotyped using the sex-linked isocitrate dehyrogenase marker, which identifies both males and females with >99% precision (Peichel et al., 2004). Polymerase chain reaction (PCR) was carried out using stickleback specific forward (STKSEXFOR 5′ GGGACGAGCAAGATTTATTGG 3′) and reverse primers (STKSEXREV 5′ TATAGTTAGCCAGGAGATGG 3′) and published PCR cycle conditions (OECD, 2011a). PCR products were analysed using gel electrophoresis, and fish scored as females (which produce a single ∼300 bp PCR product) or males (which produce two products of ∼270 bp and ∼300 bp).

### 2.7 Statistical analyses

Non-normally distributed variables were identified using the Kolmogorov–Smirnov statistic and either transformed appropriately or, where transformation did not normalise the data, analysed using non-parametric statistical tests. Chi-square tests of independence were used to identify any effect of E2 treatment and sex on infection susceptibility. A non-parametric (Kruskal–Wallis) ANOVA was used to test for differences in VTG levels between E2 treatments. General Linear Models (GLMs) were used to test the effect of E2 treatment, host sex and infection status (all modelled as fixed factors), and tank (nested within treatment, modelled as a random factor) on VTG level and body condition. Regression analyses revealed a significant relationship between fish length and parasite mass (regression; *F*_1,90_ = 8.3, *r*^2^ = 0.084, *P* = 0.005), so residuals were first calculated before testing the effect of host sex and E2 treatment using two-way type II ANOVA. ANCOVA was used to test for sex differences in the relationship between tissue VTG levels and residual plerocercoid mass.

Where possible, the aquarium in which fish were housed (‘tank’) was included as a random factor in statistical models. However, as there were only three instances in which multiple male and female fish were co-located in the same tank (i.e. 24 of the 27 tanks included either 0 or 1 infected fish of one sex), including ‘tank’ as a random factor was not possible in some analyses, for example when examining sex differences in residual plerocercoid mass. In these cases, infected fish are treated as statistically independent units in our analyses.

### 2.8 Ethical note

Laboratory studies were undertaken under the authority of a U.K. Home Office licence, in accordance with local and national regulations, and in line with ABS/ASAB guidelines for the ethical treatment of animals in behavioural research (http://asab.nottingham.ac.uk/ethics/guidelines.php). Infected fish were monitored closely throughout the study to ensure that no fish exhibited signs of obvious distress or lasting harm, and U.K. Home Office approved (Schedule 1) methods of euthanasia were adopted.

## 3 Results

### 3.1 Effect of E2 treatment and fish sex on infection susceptibility

Overall, 93 of the 203 surviving fish that were fed copepods containing infective procercoids developed infections (45.8%). The development of infections was not associated with E2 treatment (solvent control: 29/68; 10 ng l^−1^: 36/66; 100 ng l^−1^: 28/69; *n* = 203, *χ*^2^_2_ = 3.1, *P* = 0.216) or fish sex (females: 48/107; Males: 44/95; *n* = 202, *χ*^2^_1_ = 0.004, *P* = 0.947).

### 3.2 Effect of E2 treatment, sex and infection status on fish size and condition

**Fig. 2:**
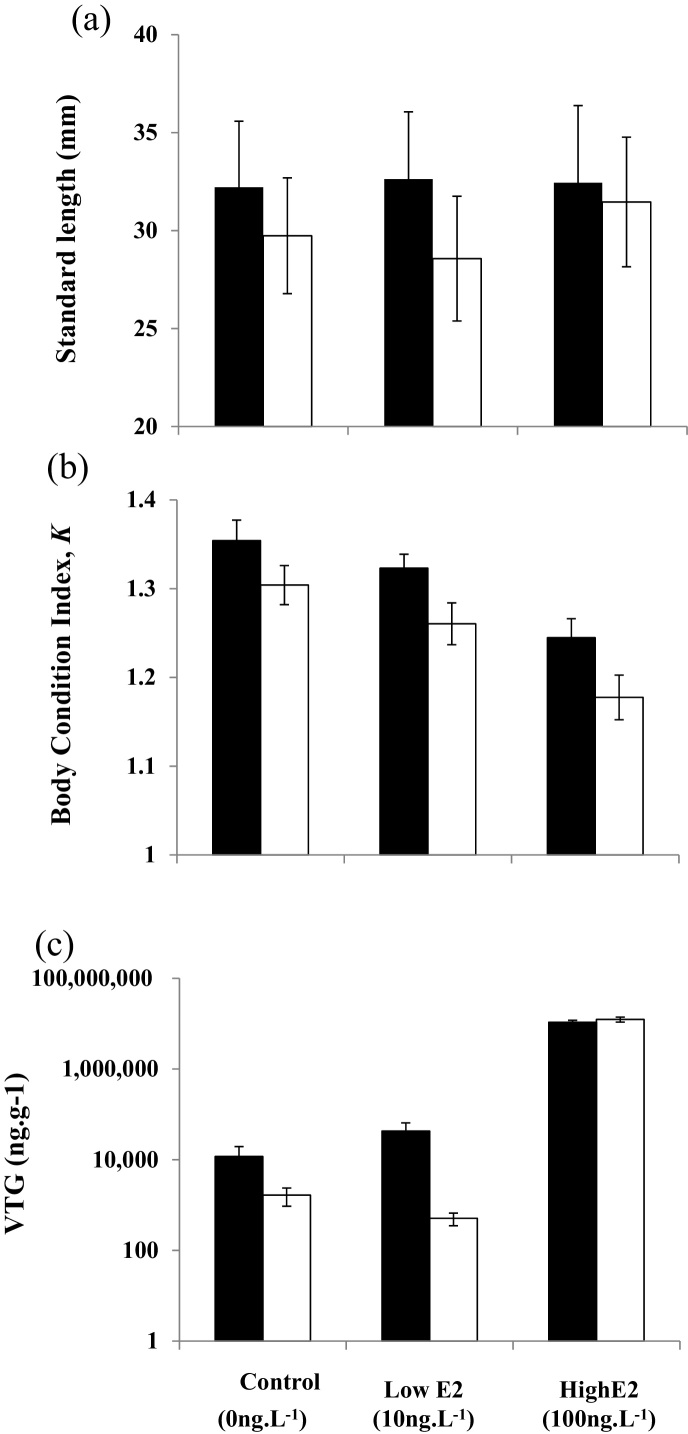
The effects of fish sex and E2 treatment on (a) standard length, (b) body condition and (c) tissue VTG level. Females: filled bars; males: open bars. Bar heights show mean values, error bars represent ± 1SD. See text and Table 1 for details of statistical tests.

Treatment with E2 had no effect on terminal body size, nor did infection status, but there was a significant effect of sex, with females being larger than males (Fig. 2a, Table 1). There was no main effect of infection status on fish body condition factor (*K*) at the end of the study; however *K* was significantly affected by both fish sex and by E2 treatment, with females having higher *K* values than males, and with *K* being reduced with increasing E2 dose (Fig. 2b; Table 1). There was a marginally significant interaction between E2 treatment and infection status on *K*, with non-infected, solvent-control fish having the highest *K* values. There was also a significant tank effect, indicating greater similarity in *K* values among fish within tanks than between tanks exposed to the same E2 treatment.

### 3.3 Effects of E2 treatment, sex and infection status on tissue VTG levels

Tissue VTG level was not correlated with body size in any treatment (solvent control: Pearson’s *r* = −0.072, *P* = 0.71, 10 ng l^−1^: *r* = 0.014, *P* = 0.94, 10 ng l^−1^: *r* = −0.304, *P* = 0.12). There was a highly significant effect of E2 treatment (Fig. 2c, Table 1), with fish in the high E2 group presenting higher VTG levels than those from the solvent control and low E2 groups. Tissue VTG level was not significantly affected by parasite infection status, but fish sex had a significant effect, with females exhibiting higher VTG levels than males (Fig. 2c, Table 1). A marginally significant interaction existed between E2 treatment, sex and infection status. There was a highly significant tank effect on VTG levels, indicating greater similarity in VTG levels among fish within tanks than between tanks exposed to the same E2 treatment.

### 3.4 Effect of E2 treatment and host sex on parasite size

**Fig. 3:**
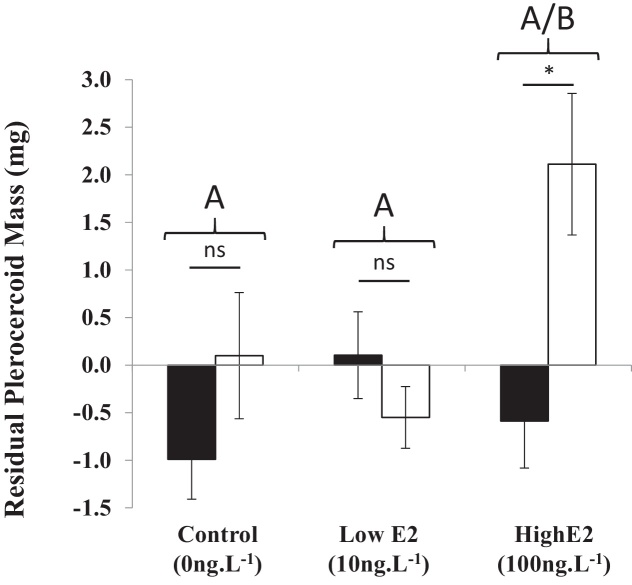
The effects of fish sex and E2 treatment on parasite mass, expressed as residuals from the relationship with fish length, in sticklebacks experimentally infected with *Schistocephalus solidus*. Females: filled bars; males: open bars. Bar heights show mean values, error bars represent ± 1SD. Capital letters (A/B) above paired bars indicate significant differences between treatment groups. Asterisks show level of significant difference between sexes (*P < 0.05; **P < 0.01; ***P < 0.005).

The residual mass of plerocercoids recovered from infected fish, after correction for host body size, was affected by a significant interaction between E2 treatment and host sex (2-way ANOVA; *F*_2,85_ = 4.1, *P* = 0.02). Whereas male and female fish in the solvent control and low E2 treatment developed parasite loads of equivalent mass, male hosts in the high E2 treatment developed significantly larger parasite loads than females (Fig. 3).

### 3.5 Relationship between VTG level and plerocercoid mass among infected fish

**Fig. 4:**
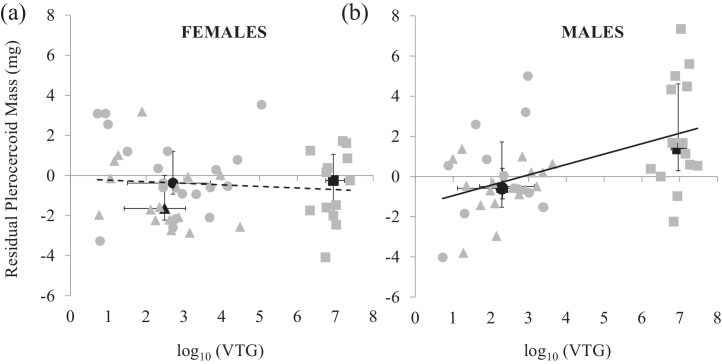
The relationship between tissue VTG levels and residual plerocercoid mass for (a) female and (b) male sticklebacks experimentally infected with *Schistocephalus solidus* and held under control (0 ng l^−1^, grey circular symbols), low E2 (10 ng l^−1^, grey triangular symbols) and high E2 treatments (100 ng l^−1^, grey square symbols). Median ± interquartile ranges for each treatment group are also shown (black symbols).

Combining data from all experimentally infected fish in the study permitted a statistical analyses of the relationship between tissue VTG levels and parasite growth, expressed as residual plerocercoid mass from the relationship with host body size. ANCOVA showed that although a significant relationship existed between tissue VTG and residual plerocercoid mass across the whole sample (*F*_1,91_ = 5.46, *P* = 0.022), a highly significant interaction between the effects of host sex and tissue VTG levels indicated that the slope of the relationship for males and females differed (*F*_1,91_ = 10.08, *P* = 0.002; Fig. 4). Separate regressions subsequently run on the male and female datasets demonstrated a significant relationship only among male fish (*F*_1,33_ = 13.33, *r* = 0.241, *P* = 0.001) and not among females (*F*_1,47_ = 0.40, *r* = 0.008, *P* = 0.53), indicating that plerocercoid growth and VTG levels were only correlated among E2 exposed male hosts (Fig. 4).

## 4 Discussion

**Fig. 5:**
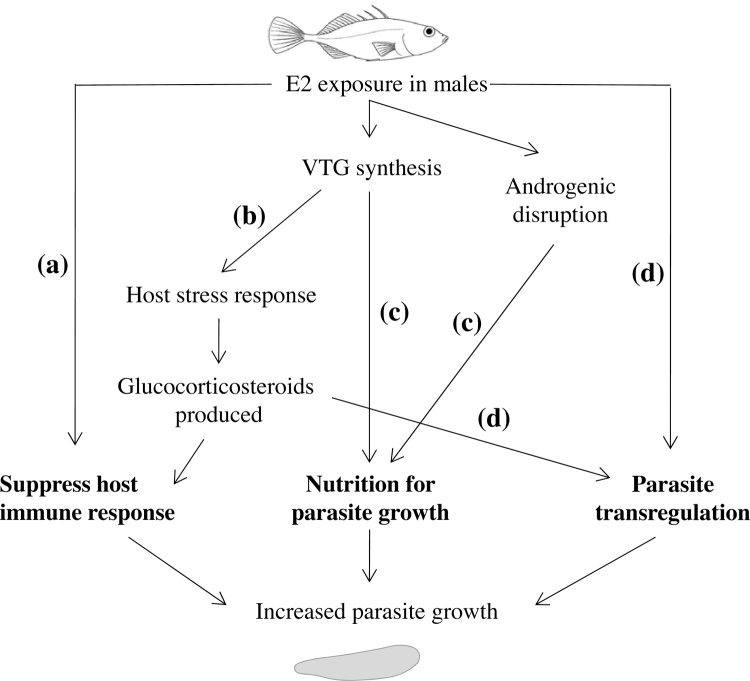
Schematic diagram illustrating four non-exclusive immune-physiological mechanisms that could be responsible for the observed increased rates of parasite growth in male host fish held under the high E2 treatment: (a) immunosuppressive effects of oestrogenic pollutants; (b) pathological effects of vitellogenin production; (c) changes in nutrient availability, either as a result of VTG being used directly as a substrate by parasites, or as a consequence of altered male hormone levels; (d) parasite transregulation. See Section 4 for further explanation.

Anthropogenic chemical contaminants in general, and endocrine disruptors in particular, pose a major threat to the health of aquatic ecosystems (Jobling et al., 1998, Kidd et al., 2007, Tyler et al., 1998). Yet despite an improving understanding of the effects on individual organisms, the potential for pollutants to influence ecological interactions between species is far less well understood. One area of serious concern relates to the potential of anthropogenic chemicals to exacerbate the effects of naturally occurring pathogens and disease causing agents, leading to effects on disease prevalence and infection intensity (Lafferty and Kuris, 1999, Lafferty and Kuris, 2005). For example, laboratory and field experiments have demonstrated that the exposure of amphibians to pesticides resulted in increased infection levels with parasitic trematodes, and these stressors applied in combination have a synergistic effect on the development of limb deformities (Kiesecker, 2002). In the present study we experimentally exposed stickleback fish to controlled doses of a naturally occurring parasite, *S. solidus*, in the presence or absence of a model oestrogen, 17β-estradiol (E2). Although E2 exposure did not significantly affect the probability that sticklebacks developed infections after ingesting infective stages of the parasite, the rate of disease progression—measured as the growth of plerocercoids—was significantly faster in male fish from the high E2 treatment. These effects of E2 on parasite growth emerged despite a small absolute plerocercoid mass after 28 days growth, in comparison to earlier studies employing longer post-exposure periods (Barber and Svensson, 2003, Macnab and Barber, 2012). Because the time taken for individual plerocercoids to achieve infective mass reflects early growth trajectories and determines the mass ultimately achieved in the fish (Barber and Svensson, 2003), our results suggest that the effects of EDCs on early plerocercoid growth may shorten parasite life cycles and/or increase parasite fecundity in the adult host (Fig. 5).

To our knowledge, these results represent the first experimental demonstration of the effects of an oestrogen on parasite growth in a fish. In this host–parasite system, the size attained by plerocercoids in the fish host determines the phenotypic effects of infection, with impacts on ecologically-important host traits—including gonad development, reproductive behaviour, antipredator responses and the ability to withstand further environmental stress—all being more severe in fish harbouring larger parasites (Barber et al., 2004, Heins et al., 2010, Macnab et al., 2011). Hence, environmental changes that increase parasite growth rates speed up the progression of disease in infected fish, with implications for the rate and nature of interactions with predators, potential mates and competitors. Our finding that parasitized males are disproportionately affected by EDC exposure additionally suggests that the effects of pollutants on host–parasite interactions may be complex, and depend on the sex (or other characteristics) of the host. For male sticklebacks, *S. solidus* infection is typically associated with compromised sexual development, nesting behaviour and courtship, with these effects being driven by depressed 11-ketotestosterone titres (Macnab et al., 2011); however, these effects are closely related to the size attained by the *S. solidus* plerocercoids, with males harbouring smaller worms being capable of reproductive development and behaviour (Macnab et al., 2009, Macnab et al., 2011). Our results therefore suggest that in polluted environments with high oestrogenic burden—which may already be detrimental to sexual development—increased parasite growth may further limit the reproductive capacity of infected males, and generate skew in the operational sex ratio.

Our results also have implications for the timing and frequency of parasite life cycle completion in perturbed environments, since the infectivity of *S. solidus* to bird hosts (Tierney and Crompton, 1992), the parasite-manipulated stickleback behaviour that facilitates transmission (Barber et al., 2004) and the fecundity of adult worms in the definitive host (Dörücü et al., 2007) all correlate strongly with the mass attained by plerocercoids in the stickleback host. In environments that enhance plerocercoid growth, a greater proportion of hosts will harbour infective plerocercoids at any given time. In waters subject to EDC pollution, our results suggest an increased frequency of successful bird predation on infected sticklebacks, leading to parasite life cycles being completed more quickly and increasing the input of infective stages into aquatic environments.

In our study, both male and female sticklebacks exhibited increased VTG production in response to E2 exposure, though the responses differed. Vitellogenin is produced in the liver of oviparous fish after stimulation from increased levels of circulating E2; this process is considered to take place naturally only in female fish, hence the presence of VTG in male blood/tissues has been used for decades as an unambiguous biomarker of exposure to xenoestrogens (Denslow et al., 1999, Kime et al., 1999, Sumpter and Jobling, 1995). However, low, biologically insignificant levels of VTG—such as those detected in our solvent control treatment—can also be registered in male fish (Hotta et al., 2003, Ma et al., 2005, Scott and Robinson, 2008), including sticklebacks (Katsiadaki et al., 2012), most likely due to the presence of endogenous low levels of E2 (Sebire et al., 2007). Aromatisation of androgens to oestrogens particularly in the brain is a well described phenomenon in vertebrates (Lephart, 1996) including fish (González and Piferrer, 2003) and importantly sticklebacks (Borg et al., 1989, Borg et al., 1987a, Borg et al., 1987b). Whereas female fish showed a graded, stepwise increase in VTG level in response to low and high E2 treatment, VTG levels among males were not elevated in response to the low E2 treatment, and only increased under the high E2 treatment. This suggests that males may have a higher E2 threshold for VTG synthesis, but above this threshold both males and females respond equally.

In our study, high levels of VTG only led to increased plerocercoid growth among infected male fish, and not among infected females. Here, we outline different types of mechanism that could be responsible for the increased parasite growth observed in male hosts held under the high E2 regime (Fig. 5).

### 4.1 Immunosuppressive effects of oestrogenic pollutants

Oestrogenic contaminants have the potential to modulate host immune responses (Milla et al., 2011) and bidirectional interactions can occur between endocrine and immune systems (Ahmed, 2000, Weyts et al., 1999). Physiological levels of E2 can have an immunosuppressive effect on goldfish (*Carassius auratus*) (Wang and Belosevic, 1994) and mechanisms can include the repression of acute phase immune response genes (Tilton et al., 2006) and reduced phagocytic activity (Milla et al., 2011, Williams et al., 2007). Such a mechanism may explain our results if high levels of E2 have a reduced immunosuppressive effect on females, which might be expected given their elevated circulating oestrogens during vitellogenesis.

### 4.2 Pathological effects of vitellogenin production

In female fish, oestrogens lead to the production of VTG in the liver, which is transported via the blood to the ovaries where it enters developing oocytes and is stored for use by developing embryos (Clemens, 1974, Purdom et al., 1994). Under normal conditions, males do not produce VTG (Folmar et al., 2001) and importantly they lack ovaries that could sequester it from the plasma; hence male fish have not evolved to utilise—and have no capacity to store—the protein (Zaroogian et al., 2001). Previous studies have shown that intensive VTG synthesis by male fish can generate adverse health effects, including renal failure due the increased need for clearance of vast amounts of protein (impaired osmoregulation) (Folmar et al., 2001, Thorpe et al., 2007, Zaroogian et al., 2001). In our study, fish in the high E2 treatment had lower condition factors than other groups, and males from the high E2 group had the lowest condition factors. VTG production may have disturbed homeostasis and induced a stress response in males (Wendelaar Bonga, 1997), with their increased parasite load arising as a consequence of the immunosuppressive action of elevated glucocorticosteroids (Wendelaar Bonga, 1997, Weyts et al., 1999).

### 4.3 Increased nutrient availability for parasite growth

Another possibility is that host-produced VTG, or its breakdown products, is used directly by developing parasites as a substrate to fuel growth. This explanation is consistent with our finding that fish exhibiting the highest VTG levels developed the largest parasites, and may also explain sex differences in parasite growth. As male fish are unable to utilise VTG by ovarian sequestration, it is likely that the accumulation of VTG in the high E2 exposed male hosts was more readily available to growing parasites. Another related possibility is that environmental oestrogens influence the synthesis of androgens in males, with consequences for male energetics that influence condition and parasite growth.

### 4.4 Parasite transregulation

Alternatively, the parasite could use the host’s hormonal environment to increase its own growth by transregulation (Escobedo et al., 2005). The effects of host sex steroids on parasite growth are often thought to be indirect, via the regulation of the immune response (Escobedo et al., 2004); however host-derived sex steroids may also affect parasites directly. E2 promotes the in vitro proliferation of *Taenia crassiceps* cysticerci, which express both ER-α and ER-β oestrogen receptors (Escobedo et al., 2004). It is possible that the hormonal environment experienced by parasites infecting fish exposed to the high E2 treatment could have a direct effect on parasite growth. Alternatively, because adrenal hormones can affect parasite growth directly (Carrero et al., 2006, Morales-Montor et al., 2001), host-derived cortisol could potentially be utilised by parasites following a stress response in males experiencing the high E2 treatment.

How relevant are our results to normal exposure levels in natural environments? Levels of E2 in the environment rarely reach 100 ng l^−1^, except for greatly perturbed ecosystems (Yang et al., 2011). However the total oestrogenic burden of aquatic bodies, expressed as ng^−1^ E2 equivalents is very often at this range particularly in river and estuarine sediments (Tollefsen and Thomas, 2006). Since fish are typically exposed simultaneously to complex mixtures of multiple pollutants (Sumpter, 2009), it is highly possible that mixtures of oestrogenic chemicals, all at lower concentrations, exert additive effects (Silva et al., 2002). Increased *S. solidus* growth is also observed in sticklebacks experiencing elevated temperatures (Macnab and Barber, 2012), so there is also the possibility for low-level oestrogenic pollution to interact with altered thermal regimes to significantly impact parasite mass and infection phenotypes. Such conditions could be encountered downstream of sewage treatment plants in the U.K., where effluent often constitutes a high proportion of flow.

In conclusion, we have shown that exposure to E2, a model oestrogen representative of the mode of action of many oestrogenic xenobiotics, significantly affected the body condition, VTG level and parasite load of sticklebacks. Importantly, our results also show that the effects of EDC pollutants on the interactions between hosts and parasites can be sex-dependent, with males developing larger plerocercoids under EDC treatment than females, and hence being more severely impacted. Our results therefore confirm the potential for environmental oestrogens to modulate ecologically relevant infection phenotypes. Further studies are now required to elucidate the precise mechanisms involved, to investigate the generality of the finding in other aquatic host–parasite systems, and develop a greater understanding of the ecological consequences of altered host–parasite interactions in anthropogenically polluted ecosystems.

## Author contributions

The study was conceived by IB and IK, and the experimental work was undertaken by VM and CT under the supervision of IB at Leicester. Data analyses was undertaken by IB and VM, with additional interpretation by IK. The manuscript was written by IB and VM with input from IK.

## Declaration

Financial support for the conduct of the research was provided by the UK BBSRC and Cefas, who were the industrial CASE partners in the project. Neither funder played any role in the collection, analyses or interpretation of data, in the writing of the report or in the decision to submit the article for publication.

## Data accessibility

All experimental data will be made publicly available following publication on the University of Leicester Research Archive (https://lra.le.ac.uk).

## Figures and Tables

**Table 1:** Effects of E2 treatment (solvent control/10 ng l^−1^/100 ng l^−1^), infection status (infected/non-infected), sex (male/female) and holding tank (27 levels, nested within E2 treatment) on fish length, condition factor (*K*) and vitellogenin (VTG) levels, estimated by General Linear Models. Interactions that could be estimated in the model are shown; other interaction terms could not be estimated are removed.

Source		Length		Condition factor (*K*)		VTG	
	d.f.	*F*	*P*	*F*	*P*	*F*	*P*
E2 treatment	2, 201	3.35	0.050	8.96	0.001	183.28	0.0005
Infection status	1, 201	0.47	0.494	0.29	0.590	0.61	0.434
Sex	1, 201	16.70	<0.0005	4.86	0.029	6.30	0.013
Tank (E2 treatment)	24,201	0.71	0.833	1.88	0.011	3.32	<0.0005
E2 treatment × infection status	2, 201	0.03	0.969	3.27	0.041	0.72	0.490
E2 treatment × sex	2, 201	1.43	0.242	0.05	0.955	2.24	0.109
Infection status × sex	1, 201	1.68	0.196	1.92	0.168	1.19	0.278
E2 treatment × infection status × sex	2, 201	0.44	0.648	1.19	0.393	3.35	0.038
